# A Hospital Fete Story

**Published:** 1920-08-28

**Authors:** 


### A Hospital Fete Story.

"PICKLES."

I readily accepted the prize, little dreaming of the uncomfortable hours which lay before me. I had entered a competition at the local fête and sports for a young "porker" ten weeks old, and, to my surprise, I found myself a pig-owner. I ruminated that I had a good stretch of garden, and that I could "do my bit" to increase the food supply by pig-keeping, which I was informed was profitable.

I started homeward to the greetings of the spectators. With sundry taps and nudges with my cane, I directed Pickles on the course I wished him to take, but I found he had a will of his own. The gate he wanted to make his exit by was not the one I selected; this was my first difficulty with him. Prod and persuade as I would, his way homeward was not mine. I concluded he was terrorised by the crowd. Pickles' way was through that crowd. I cracked my sides at the attempts of the spectators, the ladies particularly, to avoid him. The male element scented sport, and the attempts to catch Pickles only served to increase the uncertainty of his movements, and his evasions of capture.

It was a full hour before Pickles left that field. Pickles, I had come to the conclusion, was wilful—he would do everything but go home. From side to side of the road, into ditches, and more than once through hedge-gaps into fields, this was the course of his movements.

After a time I took him under my arm. He was heavy, and I did not bargain for his struggles for freedom. Besides, he really didn't smell cleanly, his habits, too, left something to be desired, and I put him down.

It was 2.30 A.M. when we arrived home. With a step-ladder, kitchen chairs, and sundry boxes bundled together in the darkness, I hoped Pickles was secure. I explained, so far as dignity would allow, the cause of my late arrival to my wife, who coldly replied that a small gratuity to our butcher boy would have saved me the explanation.

In the early morning, at five, my children excitedly urged me to expel an intruder from the garden. Consoling myself with the reflection that I would take the first steps to make him a permanent stye, I hastily dressed, and, explaining the circumstances, I proceeded to hunt for Pickles. I found he had helped himself freely to my bed of winter cabbages, had sampled and trodden down my lettuces, had partly uprooted my potatoes, and generally enjoyed an unarranged picnic. We fixed him up in quarters, and my family are now hoping that Pickles will prove a real pickle in the winter months.

